# Intracardiac metastasis of follicular thyroid carcinoma

**DOI:** 10.1093/jscr/rjaf220

**Published:** 2025-07-10

**Authors:** Henrique Coutinho, Gustavo Kikuta, Bernardo Brasil, Luis Felipe Okida, Joaquim Henrique Coutinho

**Affiliations:** Department of Cardiovascular Surgery, Pedro Ernesto University Hospital, State University of Rio de Janeiro, Boulevard 28 de Setembro, 77, Vila Isabel, Rio de Janeiro, RJ 20551-030, Brazil; Department of Cardiovascular Surgery, Serra dos Órgãos University Center, Av Delfim Moreira, 2211, vale do Paraíso, Teresópolis, Rio de Janeiro 25976-016, Brazil; Department of Cardiovascular Surgery, Pedro Ernesto University Hospital, State University of Rio de Janeiro, Boulevard 28 de Setembro, 77, Vila Isabel, Rio de Janeiro, RJ 20551-030, Brazil; Department of Cardiovascular Surgery, Serra dos Órgãos University Center, Av Delfim Moreira, 2211, vale do Paraíso, Teresópolis, Rio de Janeiro 25976-016, Brazil; Department of Cardiovascular Surgery, Pedro Ernesto University Hospital, State University of Rio de Janeiro, Boulevard 28 de Setembro, 77, Vila Isabel, Rio de Janeiro, RJ 20551-030, Brazil; Department of Cardiovascular Surgery, Pedro Ernesto University Hospital, State University of Rio de Janeiro, Boulevard 28 de Setembro, 77, Vila Isabel, Rio de Janeiro, RJ 20551-030, Brazil; Department of Cardiovascular Surgery, Pedro Ernesto University Hospital, State University of Rio de Janeiro, Boulevard 28 de Setembro, 77, Vila Isabel, Rio de Janeiro, RJ 20551-030, Brazil; Department of Cardiovascular Surgery, Serra dos Órgãos University Center, Av Delfim Moreira, 2211, vale do Paraíso, Teresópolis, Rio de Janeiro 25976-016, Brazil

**Keywords:** heart tumor, follicular carcinoma methastasis, intracardiac mass, cardiovascular surgery, thyroid tumor methastasis

## Abstract

Thyroid tumors represent approximately 1% of neoplasms diagnosed in Brazil, according to data from the National Cancer Institute. Most cases are differentiated carcinomas, with only 5% to 10% producing distant metastases. Follicular carcinoma, the second most common subtype, is known for its angioinvasive characteristics, typically observed microscopically and locally. Extension to large vessels and the heart is exceedingly rare, with only a few cases reported in the literature. This aggressive form of the disease often presents with exuberant symptoms, primarily superior vena cava syndrome and right ventricular outflow tract obstruction. The hematogenous spread characteristic of follicular carcinoma increases the likelihood of distant metastases, though cardiac involvement remains uncommon. Recent studies have suggested that aggressive surgical intervention, when feasible, can significantly improve outcomes in these rare cases. This report illustrates a female patient with asymptomatic intracardiac follicular thyroid carcinoma who underwent successful surgical resection.

## Introduction

Thyroid cancer is a relatively common endocrine malignancy, accounting for 1% to 5% of all malignancies worldwide. Differentiated thyroid cancers (DTC), which include papillary and follicular thyroid carcinoma (FTC), generally have a favorable prognosis, particularly when detected early and treated appropriately [1]. While papillary thyroid carcinoma (PTC) is the most frequent histological subtype, representing up to 80% of cases, FTC accounts for approximately 10% of thyroid cancers and is characterized by its propensity for hematogenous dissemination rather than lymphatic spread [2].

FTC is well known for its angioinvasive nature, often leading to distant metastases involving the lungs and bones. However, metastasis to the heart is exceedingly rare, with a reported incidence of intracardiac involvement ranging from 0.1% to 2% in autopsy studies [3, 4]. The heart can be affected through direct tumor extension via the great veins or hematogenous spread, and such cases are associated with significant morbidity and mortality due to complications like superior vena cava syndrome, right ventricular outflow tract obstruction, and embolization [5, 6]. Cardiac metastases from thyroid carcinoma typically occur late in the course of the disease, but early detection remains critical as highlighted by several case reports [7, 8].

Several case reports and small case series have documented successful management of intracardiac metastases of FTC using a combination of surgical resection and adjuvant therapies, such as radioiodine treatment and external beam radiation therapy [9–11]. Early diagnosis through advanced imaging modalities, including transthoracic and transesophageal echocardiography, computed tomography (CT), and magnetic resonance imaging (MRI), is critical for determining the extent of disease and planning appropriate surgical intervention [12, 13]. Additionally, multimodal approaches involving endocrinologists, oncologists, and cardiovascular surgeons have been shown to improve survival outcomes [14].

Recent advances in molecular biology have identified specific genetic mutations associated with aggressive forms of FTC, such as RAS and PAX8/PPARγ rearrangements, which may contribute to its invasive behavior. These molecular markers are being investigated as potential therapeutic targets and prognostic indicators [15, 16].

## Case report

Patient M.D.P.S., female, 62 years old, homemaker, asymptomatic, with a history of total thyroidectomy and lymph node dissection in 2013 due to the diagnosis of follicular thyroid carcinoma. She remained under clinical follow-up, and in March 2017, a routine echocardiogram revealed the presence of an echogenic image measuring 2.2 × 1.2 cm in its largest diameter with significant mobility located in the right ventricular outflow tract, insinuating into the proximal segment of the pulmonary artery. Additionally, an image measuring 4.8 × 1.8 cm originating from the superior vena cava was observed, causing blood flow turbulence on Doppler and insinuating toward the tricuspid valve.

Upon admission, laboratory tests revealed normal thyroid function. Thyroglobulin levels were elevated, consistent with residual or metastatic disease. The patient was clinically stable, with no signs of superior vena cava syndrome or hemodynamic compromise. Given the extent of the tumor thrombus and its intracardiac involvement, a decision was made to proceed with surgical intervention.

**Figure 1:**
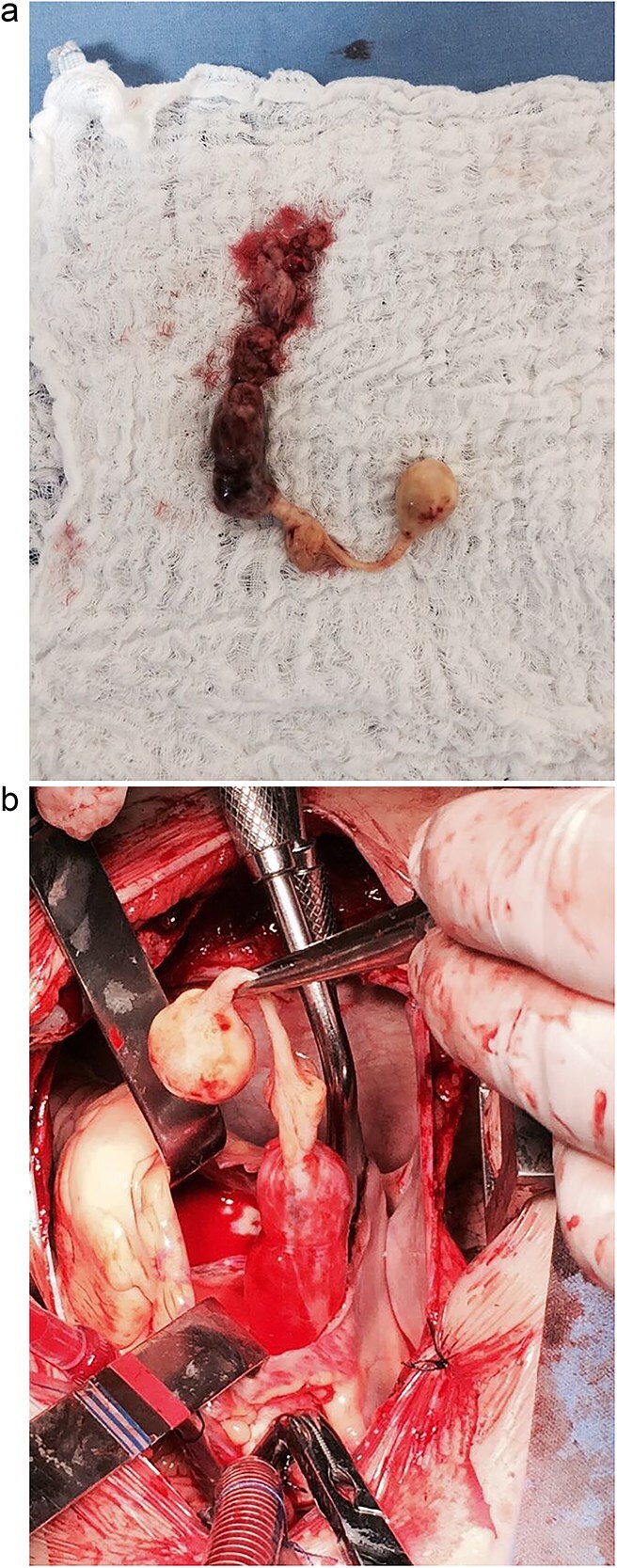
(a) Surgical view of the intracardiac tumor. (b) Intracardiac tumor as a single piece after surgical removal.

The patient was then referred to the cardiovascular surgery department, where she was admitted and underwent surgical resection on 12 April 2017. During the procedure, the patient was placed on cardiopulmonary bypass, and after right atriotomy, an elongated neoplastic-looking mass was observed, insinuating through the superior vena cava with extension to the tricuspid valve, right ventricle, and pulmonary artery infundibulum. It was necessary to extend the incision into the superior vena cava and perform phlebotomy of the left innominate vein, where the mass was adherent. Venous wall resection attached to the tumor was performed, followed by reconstruction using autologous pericardium. A single piece approximately 10 cm in length was removed and sent for pathological examination (Fig. 1). Intraoperative transesophageal echocardiography confirmed the complete removal of the mass without residual thrombus.

Postoperative management included intensive care monitoring and early mobilization. The patient received prophylactic anticoagulation and was started on levothyroxine therapy to maintain TSH suppression. The postoperative period was uneventful, and the patient was discharged on the sixth postoperative day. She was referred to the endocrinology clinic, where she continues to be followed in conjunction with clinical oncology. Histopathological examination confirmed metastatic follicular thyroid carcinoma with extensive vascular invasion.

During the follow-up period, the patient underwent periodic imaging studies, including echocardiograms and CT scans, which showed no evidence of recurrence. Thyroglobulin levels were monitored regularly as a tumor marker and remained within acceptable limits, suggesting effective disease control.

## Discussion

Intracardiac metastasis of thyroid carcinomas is a rare entity often associated with a poor prognosis due to the potential for mechanical obstruction and embolization [1, 2]. Studies emphasize the importance of a multimodal approach, including imaging diagnosis, surgical resection, and complementary therapy, such as radioiodine therapy [3, 4]. Transthoracic and transesophageal echocardiography play a crucial role in diagnosing and assessing the extent of the tumor thrombus [5].

Despite the limited literature, reports indicate that aggressive surgery can improve survival and functional outcomes [9, 10]. Successful outcomes have been reported in cases where tumor thrombi in great veins and the heart were completely resected, followed by adjuvant radioiodine therapy [7, 17]. Furthermore, it is important to differentiate between intraluminal and extraluminal tumor invasion, as the former allows for more conservative surgical approaches when feasible [18, 19].

Advancements in imaging technology have significantly improved the diagnostic accuracy for detecting cardiac involvement in thyroid cancer. Cardiac MRI, in particular, has proven useful in delineating the extent of myocardial and pericardial involvement, aiding in preoperative planning [15]. Additionally, positron emission tomography (PET) scans have been employed in select cases to assess metastatic spread and guide therapeutic decisions [16].

Multidisciplinary management involving endocrinology, oncology, and cardiovascular surgery teams is critical in ensuring optimal outcomes for such complex cases [15]. Postoperative surveillance is also paramount to detect possible recurrence or metastases, as highlighted in long-term follow-up studies [8, 16]. This case reinforces the need for strict surveillance and multidisciplinary management for patients with follicular thyroid carcinoma, especially in the presence of extensive vascular invasion.

Emerging therapies, such as targeted molecular treatments and immune checkpoint inhibitors, hold promise in managing advanced or recurrent thyroid cancers with intracardiac metastases. Clinical trials are ongoing to evaluate the efficacy of these novel agents in improving survival and quality of life [20, 21].

The role of genetic profiling in guiding therapeutic decisions is also gaining prominence. Mutations in the TERT promoter and BRAF gene, although more common in PTC, have been identified in aggressive cases of FTC. Understanding these genetic alterations may pave the way for personalized treatment approaches [22].

## Conclusion

Surgical resection of intracardiac tumor thrombi in patients with follicular thyroid carcinoma is feasible and can be performed with low complication rates in specialized centers. As highlighted in the literature, complete surgical removal of tumor thrombi, when combined with appropriate adjuvant therapies, can lead to significantly improved survival and quality of life [4, 9]. Early detection through vigilant follow-up and imaging in high-risk patients is paramount. Additionally, a multidisciplinary approach involving endocrinologists, oncologists, cardiologists, and cardiovascular surgeons is essential for optimizing treatment outcomes in such complex cases. Long-term surveillance and timely management of recurrences remain critical to ensuring prolonged survival and improved quality of life in these patients.

Furthermore, future research should focus on the development of standardized protocols for the management of intracardiac metastases in thyroid cancer. Collaborative efforts in the form of registries and multicenter studies are needed to gather robust data on long-term outcomes and the role of emerging therapies. This report underscores the importance of considering intracardiac metastasis in differential diagnoses of cardiac masses in patients with a history of thyroid cancer.

## References

[1] Manik G, Jose J, Hygriv Rao B. Follicular thyroid carcinoma with tumour thrombus extending into superior vena cava and right atrium – a case report. Indian Heart J 2016;68:S146–7. 10.1016/j.ihj.2016.05.016. 27751268 PMC5067792

[2] Lad PP, Kumar J, Sarvadnya J, et al. Staged surgical management of follicular thyroid carcinoma with extensive thrombus reaching up to right atrium. Int J Surg Case Rep 2020;66:48–52. 10.1016/j.ijscr.2019.10.050. 31805428 PMC6909162

[3] Giuffrida D, Gharib H. Cardiac metastasis from primary anaplastic thyroid carcinoma. Endocr Relat Cancer 2001;8:71–3. 10.1677/erc.0.0080071. 11350728

[4] Chiofalo MG, D’Anna R, di Gennaro F, et al. Great veins invasion in follicular thyroid cancer: single-centre study assessing prevalence and clinical outcome. Endocrine 2018;62:71–5. 10.1007/s12020-018-1622-4. 29749566

[5] Catford SR, Lee KT, Pace MD, et al. Cardiac metastasis from thyroid carcinoma. Thyroid 2011;21:855–66. 10.1089/thy.2010.0273. 21751883

[6] de Lima PRL, Crotti PLR. Malignant cardiac tumors. Rev Bras Cir Cardiovasc 2004;19:39–46.

[7] Rocha RM, MCLFS S, Musso C, et al. Well-differentiated thyroid carcinoma: epidemiological profile, surgical results, and oncological response. Rev Col Bras Cir 2018;45:e1658. 10.1590/0100-6991e-20181934 30365694

[8] Thompson NW, Dunn EL, Grant CS. Thyroid carcinoma with intracardiac extension. Am J Surg 1978;136:666–81.

[9] Kasprzak JD, Religa W, Krzemiñska-Pakula M, et al. Right ventricular outflow tract obstruction by cardiac metastasis as the first manifestation of follicular thyroid carcinoma. J Am Soc Echocardiogr 1996;9:733–5. 10.1016/S0894-7317(96)90075-9. 8887882

[10] Bussani R, De-Giorgio F, Abbate A, et al. Cardiac metastases. J Clin Pathol 2007;60:27–34. 10.1136/jcp.2005.035105. 17098886 PMC1860601

[11] Uchikawa Y, Ueno T, Ozaki M, et al. Rare presentation of cardiac metastasis in thyroid carcinoma. J Cardiothorac Surg 2015;10:113.

[12] Onoda N, Nakamura M, Hosono M, et al. Successful surgical treatment of advanced follicular thyroid carcinoma with tumor thrombus infiltrating the superior vena cava: report of a case. Surg Today 2012;42:185–90. 10.1007/s00595-011-0033-4. 22072150

[13] Wippermann J, Böning A, Habermehl P, et al. Radical reoperation for cardiac leiomyosarcoma. J Thorac Cardiovasc Surg 2010;139:e99–101.

[14] Fukuoka O, Terao Y, Nakao K, et al. Multimodal management of cardiac metastases from thyroid carcinoma. J Clin Endocrinol Metab 2014;99:3954–8.

[15] Sugishita Y, Kammori M, Yamada O, et al. Biological differential diagnosis of follicular thyroid tumor and Hürthle cell tumor on the basis of telomere length and hTERT expression. *Ann Surg Oncol* 2014;21:2318–25. 10.1245/s10434-014-3552-6. 24562933

[16] Luster M, Aktolun C, Amendoeira I, et al. European perspective on 131I therapy of thyroid cancer: the 2020 consensus report of the European Thyroid Association. *Eur J Endocrinol* 2020;183:R189–210.

[17] Elsamna ST, Suri P, Mir GS, Roden DF, Paskhover B. The benefit of primary tumor surgical resection in distant metastatic carcinomas of the thyroid. *Laryngoscope* 2021;131:1026–34. 10.1002/lary.29053. 32865854

[18] Chen J, Zhu J, Zhang C, et al. Contrast-enhanced ultrasound for the characterization of portal vein thrombosis vs tumor-in-vein in HCC patients: a systematic review and meta-analysis. *Eur Radiol* 2020;30:2871–80. 10.1007/s00330-019-06649-z. 32020403 PMC7160216

[19] Ríos A, Rodríguez JM, Ferri B, et al. Prognostic factors of follicular thyroid carcinoma. *Endocrinol Nutr* 2015;62:11–18. English, Spanish. 10.1016/j.endonu.2014.06.006 25156926

[20] Schlumberger M, Tahara M, Wirth LJ, et al. Lenvatinib versus placebo in radioiodine-refractory thyroid cancer. *N Engl J Med* 2015;372:621–30. 10.1056/NEJMoa1406470. 25671254

[21] Cabanillas ME, Ferrarotto R, Garden AS, et al. Neoadjuvant BRAF- and MEK-targeted therapy for locally advanced, BRAF V600E-mutant anaplastic thyroid cancer. *Thyroid* 2017;27:945–51.

[22] Liu R, Xing M. TERT promoter mutations in thyroid cancer. *Endocr Relat Cancer* 2016;23:R143–55. 10.1530/ERC-15-0533. 26733501 PMC4750651

